# The inhibitory effects of *Geranium thunbergii* on interferon-γ- and LPS-induced inflammatory responses are mediated by Nrf2 activation

**DOI:** 10.3892/ijmm.2015.2128

**Published:** 2015-03-06

**Authors:** HEE-JIN CHOI, HEE-JUNG CHOI, MI-JU PARK, JI-YEON LEE, SEUNG-IL JEONG, SEONGOO LEE, KYUN HA KIM, MYUNGSOO JOO, HAN-SOL JEONG, JAI-EUN KIM, KI-TAE HA

**Affiliations:** 1 Department of Pathology, College of Korean Medicine, Dongguk University, Siksa-dong, Ilsan, Gyeonggi-do, Republic of Korea; 2 Department of Korean Medical Science, School of Korean Medicine and Korean Medicine Research Center for Healthy Aging, Pusan National University, Mulgeum-eup, Yangsan, Gyeongsangnam-do, Republic of Korea; 3 Jeonju Biomaterials Institute, Jeonju, Republic of Korea; 4 Department of Korean Pathology, College of Korean Medicine, Sangji University, Wonju, Gangwon, Republic of Korea

**Keywords:** *Geranium thunbergii*, inflammation, interferon-γ, lipopolysaccharide, nuclear factor erythroid 2-related factor 2

## Abstract

*Geranium thunbergii* Sieb. et Zucc. (GT; which belongs to the Geraniaceae family) has been used as a traditional medicine in East Asia for the treatment of inflammatory diseases, including arthritis and diarrhea. However, the underlying mechanisms of the anti-inflammatory effects of GT remain poorly understood. In the present study, we examined the mechanisms responsible for the anti-inflammatory activity of GT in macrophages. The results revealed that GT significantly inhibited the lipopolysaccharide (LPS)- and interferon-γ (IFN-γ)-induced expression of pro-inflammatory genes, such as inducible nitric oxide synthase, tumor necrosis factor-α and interleukin-1β, as shown by RT-PCR. However, the inhibitory effects of GT on LPS- and IFN-γ-induced inflammation were associated with an enhanced nuclear factor erythroid 2-related factor 2 (Nrf2) activity, but not with the suppression of nuclear factor (NF)-κB activity, as shown by western blot analysis. In addition, in bone marrow-derived macrophages (BMDM) isolated from Nrf2 knockout mice, GT did not exert any inhibitory effect on the LPS- and IFN-γ-induced inflammation. Taken together, our findings indicate that the anti-inflammatory effects of GT may be associated with the activation of Nrf2, an anti-inflammatory transcription factor.

## Introduction

Inflammation is an essential part of innate immunity that protects the host from exogenous pathogens (1). However, uncontrolled inflammation is closely associated with tissue damage, and consequently causes diseases with uncontrolled inflammation (2). Therefore, the regulation of inflammatory responses remains a challenge. Among the various types of cells comprising the innate immune system, macrophages play central roles in the initiation and resolution of inflammation, mainly through phagocytosis, the production of reactive oxygen species (ROS) and the release of inflammatory cytokines (3). Thus, the suppression of macrophage activation is considered an important strategy for the treatment of these inflammatory diseases (4).

Nuclear factor erythroid 2-related factor 2 (Nrf2) is a key transcription factor that regulates the expression of various phase II detoxifying and antioxidant genes, including glutamate-cysteine ligase catalytic subunit (GCLC), NAD(P)H: quinine oxidoreductase 1 (NQO1) and heme oxygenase-1 (HO-1) (5). Under normal conditions, Nrf2 is retained in the cytoplasm in low abundance. When Nrf2 is activated by external stimuli, such as oxidative stress, electrophiles and chemical activators, it translocates to the nucleus and binds to its *cis*-acting antioxidant response element (ARE) sequence, and induces the expression of cytoprotective proteins (6). In addition, Nrf2 seems to play an important role in ameliorating inflammation. The genetic ablation of Nrf2 in knockout mice has been shown to lead to a significantly increased susceptibility to a variety of inflammatory diseases, such as acute lung injury, inflammatory bowel disease and rheumatoid arthritis (7–9). Thus, Nrf2 may be a therapeutic target for regulating these inflammatory diseases.

Herbal extracts have long been prescribed for the treatment of a variety of inflammatory diseases in Asian countries (10). Among the traditional herbal medicines, several herbs such as *Salvia miltiorrhiza* (11), *Amomum compactum* (12), *Gleditsia sinensis* (13) and *Alisma orientale* (14) have been found to suppress inflammation by activating Nrf2. In our ongoing effort to find novel candidate agents for the activation of Nrf2, we found that *Geranium thunbergii* Sieb. et Zucc. (GT; which belongs to the Geraniaceae family), known as Ijilpul in Korean, Gennoshoko in Japanese and Oriental geranium in English, enhances the transcriptional activity of Nrf2. GT is a medicinal herb used for the treatment of diverse diseases, including arthritis (15), hemorrhaging, infection (16), diarrhea and dysentery (16). Previous studies have revealed that the extract of the GT whole plant has anti-mutagenic (17), antioxidant (18), anti-obesity (19) and anti-inflammatory activities (15,20). In addition, phytochemical studies on GT have reported the extraction of tannins, lignans and flavonoids, such as geraniin, corilagin, ellagic acid, gallic acid, quercetin, kaempferol, kobusin, 4-hydroxykobusin and 7,7′-dihydroxybursehernin (16,21–23).

In this study, we investigated the inhibitory effects of GT on inflammatory responses elicited by interferon-γ (IFN-γ) and bacterial lipopolysaccharides (LPS). In addition, we determined whether these anti-inflammatory effects are dependent on Nrf2 activation using bone marrow-derived macrophages (BMDM) obtained from wild-type and Nrf2 knockout mice. The results revealed that GT suppressed the inflammatory response through Nrf2-dependent mechanisms.

## Materials and methods

### Preparation of GT extracts

Whole plants of GT, which were grown and collected in Gyeongsangbuk-do province in Korea in 2011, were purchased from Omniherb Co. (Daegu, Korea) and authenticated by Professor S. Lee at the College of Korean Medicine, Sangji University, Wonju, Korea. A voucher specimen (no. sjomph003) is kept at the College of Korean Medicine, Sangji University. The air-dried whole plant of GT (50 g) was cut and extracted with 50% EtOH (500 ml, twice) at 60°C for 3 h, assisted by ultrasonic waves (40 kHz). The extract was filtered with filter paper (6 *μ*m; Whatman PLC, Kent, UK) and concentrated using a rotary evaporator (Eyela, Tokyo, Japan). Subsequently, the extract was lypophilized using a freeze dryer (Labconco, Kansas City, MO, USA) to yield 14.2 g of powder (28.4%). The powder was dissolved in distilled water for stock solution (1 mg/ml) and diluted with culture medium prior to use in the experiments.

### Reagents and antibodies

TLR4-specific *E. coli* LPS was purchased from Alexis Biochemicals (San Diego, CA, USA). Mouse INF-γ was supplied by R&D Systems (Minneapolis, MN, USA). All antibodies used in this study, including antibodies to Nrf2 (SC-13032), the p65 subunit of nuclear factor-κB (NF-κB; SC-8008), lamin B (SC-365962) and hnRNP (SC-10030R) were from Santa Cruz Biotechnology, Inc. (Santa Cruz, CA, USA). All chemicals and reagents, including 3-(4,5-dimethylthiazol-2-yl)-2,5-diphenyltetrazolium bromide (MTT), sulforaphane (SFN) and N-acetyl cysteine (NAC) were obtained from Sigma-Aldrich (St. Louis, MO, USA) unless indicated otherwise.

### High performance liquid chromatography (HPLC) analysis of GT

HPLC analysis was performed using an Agilent 1200 series system (Agilent Technologies, Santa Clara, CA, USA), which consisted of a solvent delivery unit, an on-line degasser, a column oven, an autosampler and a multi-wavelength detector. For data analysis, LC solution software (version 1.24) was used. The analytical column used was a Phenomenex Gemini NX-C18 column (Phenomenex Inc., Torrance, CA, USA; 4.6×250 mm; pore size, 3.5 *μ*m). The mobile phases were solvent A [0.1% formic acid aqueous (v/v)] and solvent B (acetonitrile). The gradient flow was as follows: (A)/(B) = 95/5 (0 min) → (A)/(B) = 30/70 (60 min). The column temperature was maintained at 35°C. The analysis was carried out at a flow rate of 0.6 ml/min with detection at 275 nm. The column injection volume was 10 *μ*l. A standard solution, containing geraniin (ChemFaces, Wuhan, China) and quercetin (Sigma-Aldrich), was prepared by dissolving in distilled water (10 mg/100 ml). The solution was filtered through a 0.45 *μ*m membrane filter and HPLC was performed.

### Mice

Male C57BL/6 mice, inbred in a specific pathogen-free facility, were purchased from Samtako Bio Korea, Ltd. (Osan, Korea). Nrf2 knockout mice, which were generated from C57BL/6 mice, were a generous gifted from Dr Jefferson Chan (Pathology and Laboratory Medicine, University of California, Irvine, CA, USA). The animals were housed in certified, standard laboratory cages, and allowed access to food and water *ad libitum* prior to use in the experiments. All experimental procedures followed the Guidelines for the Care and Use of Laboratory Animals of the National Institutes of Health of Korea, and all the experiments were approved by the Institutional Animal Care and Use Committee of Pusan National University, Pusan, Korea.

### Cell culture

RAW264.7 cells (American Type Culture Collection, Rockville, MD, USA) were cultured in Dulbecco’s modified Eagle’s medium (DMEM; Invitrogen, Carlsbad, CA, USA) containing *L*-glutamine (200 mg/l; Invitrogen) supplemented with 10% (v/v) heat-inactivated fetal bovine serum (FBS; Sigma-Aldrich), 100 U/ml penicillin and 100 *μ*g/ml streptomycin (Invitrogen) and maintained in a humidified incubator at 37°C and 5% CO_2_ prior to use in the experiments.

BMDM were prepared as previously described (24). Briefly, following the asphyxiation of the mice with CO_2_, cellular material from the femurs was aspirated, pressed through a nylon mesh filter (30 *μ*m; Merck Millipore, Billerica, MA, USA), and centrifuged at 400 × g at 4°C for 5 min. Subsequently, the cells were re-suspended in DMEM containing 10% FBS, 100 U/ml penicillin, 0.1 mg/ml streptomycin, 2 mM *L*-glutamine and 50 ng/ml macrophage-colony stimulating factor (M-CSF; PeproTech, Rocky Hill, NJ, USA). Following incubation at 37°C and 5% CO_2_ for 24 h, the cells were washed 3 times with DMEM to remove non-adherent cells and cultured for 1 week, which was subsequently replaced every 2 days. The cells were then detached, washed, counted and replated for the experiments.

### Cell viability assay

The viability of the RAW264.7 cells was assessed using an MTT-based colorimetric assay. In brief, MTT solution (2.0 mg/ml) was added to each well of the cells cultured in a 96-well plate. At 4 h after incubation at 37°C in a CO_2_ cell culture incubator, the supernatants were removed and the formazan crystals formed in viable cells were measured at 540 nm using a microplate reader (VICTOR3; Perkin Elmer, Waltham, MA, USA). The percentage of living cells was calculated against the untreated cells.

### Isolation of RNA and RT-PCR

Total RNA was isolated from the tissues and cells using a Qiagen RNeasy mini kit (Qiagen, Hilden, Germany). Three micrograms of total RNA were then reverse transcribed using M-MLV reverse transcriptase (Promega, Madison, WI, USA) and single-stranded cDNA was amplified by PCR with a set of specific primers as follows: inducible nitric oxide synthase (*iNOS)* forward, 5′-CTGCAGCACTTGGATCAGGAACC-3′ and reverse, 5′-GGGAGTAGCCTGTGTGCACCTGGAA-3′; tumor necrosis factor-α (*TNF-α*) forward, 5′-CTACTCCTCAGAGCCCCCAG-3′ and reverse, 5′-AGGCAACCTGACCACTCTCC-3′; interleukin-1β (*IL-1β*) forward, 5′-GTGTCTTTCCCGTGGACCTT-3′ and reverse, 5′-TCGTTGCTTGGTTCTCCTTG-3′; *NQO1* forward, 5′-GCAGTGCTTTCCATCACCAC-3′ and reverse, 5′-TGGAGTGTGCCCAATGCTAT-3′; *HO-1* forward, 5′-TGAAGGAGGCCACCAAGGAGG-3′ and reverse, 5′-AGAGGTCACCCAGGTAGCGGG-3′; *GCLC* forward, 5′-CACTGCCAGAACACAGACCC-3′ and reverse, 5′-ATGGTCTGGCTGAGAAGCCT-3′; and glyceraldehyde-3-phosphate dehydrogenase (*GAPDH*) forward, 5′-GGAGCCAAAAGGGTCATCAT-3′ and reverse, 5′-GTGATGGCATGGACTGTGGT-3′. For PCR amplification, Taq PCRx DNA polymerase (Invitrogen) was used. The conditions used for the reaction were as follows: an initial denaturation at 95°C for 5 min followed by 22 cycles for *GAPDH*; 25 cycles for *IL-1β*, *TNF-α*, *iNOS*, *NQO1* and *HO-1*; and 30 cycles for *GCLC* of denaturation for 40 sec at 95°C, annealing for 40 sec at 57°C, and extension for 50 sec at 72°C with a final extension for 7 min at 72°C. The amplified DNA were then separated on 1.2% agarose gels in 1X TBE buffer at 100 V for 30 min, stained with ethidium bromide (Sigma-Aldrich) and visualized under UV light.

### Reporter cell lines and luciferase assay

To assay NF-κB and Nrf2 transcriptional activity, we used previously constructed reporter cell lines stably harboring an NF-κB/luciferase reporter and NQO1/luciferase reporter (25). Luciferase activity was examined using a luciferase assay kit (Promega) and normalized by the amount of total proteins from the total cell extract.

### Western blot analysis

Nuclear proteins of 5×10^6^ cells were isolated using an NE-PER nuclear extraction kit (Thermo Fisher Scientific, Waltham, MA, USA) and the amount of protein was measured using the Bradford method (Bio-Rad Protein assay; Bio-Rad Laboratories, Hercules, CA, USA). Equal amounts of proteins were electrophoresized by sodium dodecyl sulfate-polyacrylamide gel electrophoresis (SDS-PAGE) and transferred onto PVDF membranes (Bio-Rad Laboratories). The blots were blocked for 1 h with 5% non-fat dry milk prior to incubation with antibodies against Nrf2, NF-κB (p65), lamin B and hnRNP at 4°C overnight. Following incubation with secondary antibodies conjugated to HRP at room temperature for 1 h, the bands of interest were revealed by chemiluminescence (SuperSignalWest Femto; Thermo Fisher Scientific).

### Measurement of ROS production

Intracellular ROS generation was determined by 5-(and-6)-carboxy-2′,7′-dichlorodihydro-fluorescein diacetate (carboxy-H2DCFDA; Molecular Probes, Eugene, OR, USA). Briefly, the cells were treated with 100 *μ*M carboxy-H2DCFDA in culture medium and incubated at 37°C for 30 min. The cells were then washed with phosphate-buffered saline (PBS) and their fluorescence was measured using a BD FACSCanto II system (BD Biosciences, San Jose, CA, USA) at an excitation wavelength of 488 nm and an emission wavelength of 525 nm. The measured values were calculated as a percentage of the control.

### Densitometric and statistical analysis

The intensity of the bands obtained from RT-PCR and western blot analysis were quantified using ImageJ software (NIH, Bethesda, MD, USA). The values were calculated as fold increases over the control and are expressed as the means ± SD. The differences between 2 groups were determined by a one-way analysis of variance (ANOVA) with a post hoc test. The minimum significance level was set at a p-value of 0.05 for all analyses. All experiments were independently performed at least 3 times.

## Results

### GT reduces the INF-γ- and LPS-induced expression of inflammatory-related genes

**Figure 1:**
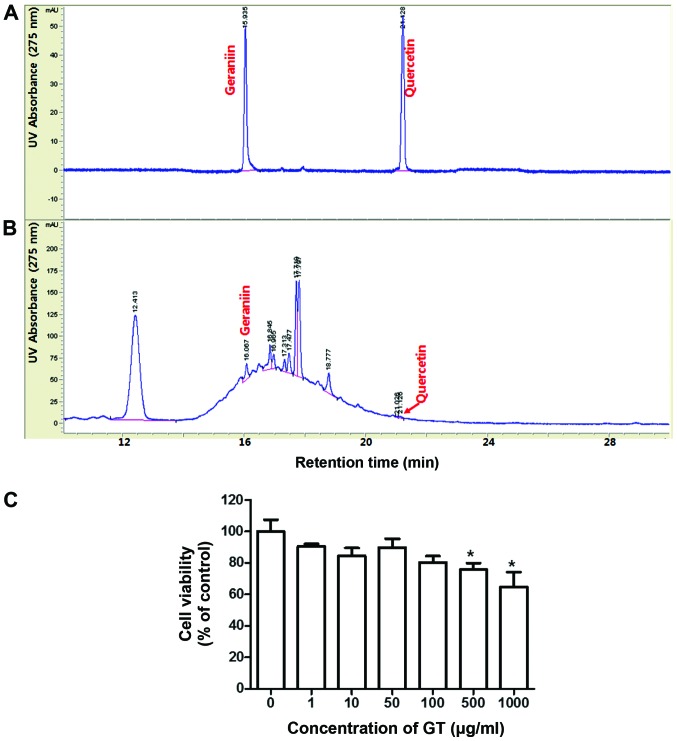
Chromatographic analysis and cytotoxic effects of *Geranium thunbergii* (GT). (A) HPLC chromatogram of mixed standard solutions, geraniin and quercetin. (B) HPLC chromatogram of 50% aquaeous-ethanol extract of GT. (C) RAW264.7 cells were treated with the indicated concentrations of GT for 24 h, and the viability of the cells was examined by MTT assay. The data from 3 independent experiments are represented as the means ± SD. ^*^p<0.05 compared to the controls.

At first, the extract was analyzed for the constituents of GT by comparing it with the known components, geraniin and quercetin (23,26) (Fig. 1A and B). Subsequently, we determined an optimal dose of GT, at which does not show any significant cytotoxicity, by MTT assay. GT showed no significant cytotoxicity to the RAW264.7 cells at concentrations of up to 100 *μ*g/ml (Fig. 1C). The half maximal inhibitory concentration (IC_50_) of GT to affect the viability of the RAW264.7 cells was >1,000 *μ*g/ml. Thus, we used GT at concentrations of up to 100 *μ*g/ml for the subsequent experiments.

**Figure 2:**
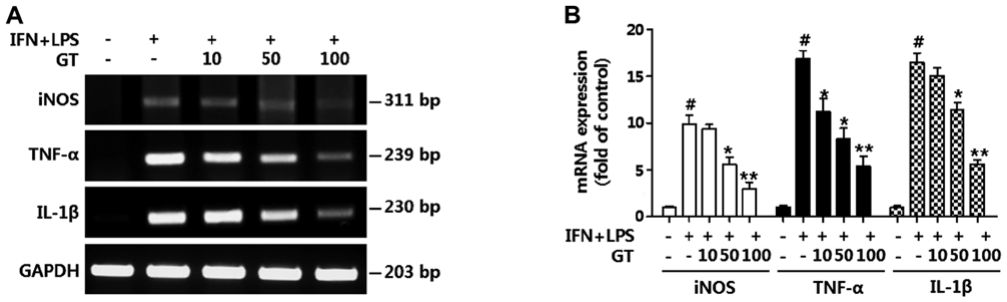
*Geranium thunbergii* (GT) suppresses the expression of pro-inflammatory genes. RAW264.7 cells were treated with the indicated concentrations of GT for 4 h, prior to stimulation with interferon-γ (IFN-γ) and lipopolysaccharides (LPS) (10 ng/ml, respectively). The cells were cultured for 16 h. (A) The expression of pro-inflammatory genes, includinginducible nitric oxide synthase (*iNOS*), tumor necrosis factor-α (*TNF-α*), and interleukin-1β (*IL-1β*) was evaluated by RT-PCR. The expression of glyceraldehyde-3-phosphate dehydrogenase (*GAPDH*) was used as an internal control. (B) The intensity of the bands was densitometrically quantified and calculated as the mean ± SD of 3 independent experiments. ^#^p<0.01 compared to the negative control (first column); ^*^p<0.05 and ^**^p<0.01 compared to the positive control (second column).

In order to examine the effects of GT on INF-γ- and LPS-induced inflammation, the cells were pre-treated with GT for 4 h prior to stimulation with INF-γ- and LPS, and the expression levels of NF-κB-dependent genes were analyzed by RT-PCR. The results revealed that GT significantly reduced the expression levels of NF-κB-dependent pro-inflammatory genes, including iNOS, TNF-α and IL-1β (Fig. 2). The IC_50_ values of GT to affect the expression of inflammatory genes were approximately 60 *μ*g/ml for iNOS, 63 *μ*g/ml for TNF-α and 75 *μ*g/ml for IL-1β.

### GT enhances Nrf2 activity, but does not suppress NF-κB activity

**Figure 3:**
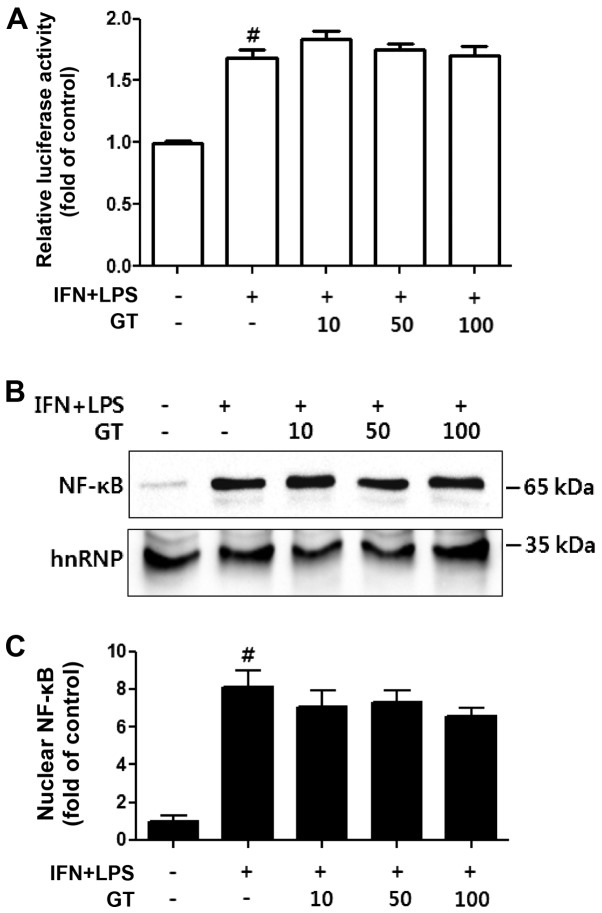
*Geranium thunbergii* (GT) does not directly inhibit the transcriptional activity and nuclear translocation of nuclear factor-κB (NF-κB) in RAW264.7 cells. (A) An NF-κB reporter cell line, derived from RAW264.7 cells, was pre-treated with the indicated concentrations of GT for 30 min, then stimulated with interferon-γ (IFN-γ) and lipopolysaccharides (LPS) (10 ng/ml, respectively) for 16 h. ^#^p<0.01 compared to the negative control (first column). (B) The amount of nuclear p65 subunit of NF-κB was examined by western blot analysis. The amount of hnRNP was used as an internal control. (C) The intensity of the bands was densitometrically quantified and calculated as the mean ± SD of 3 independent experiments. ^#^p<0.01 compared to the negative control (first column).

**Figure 4:**
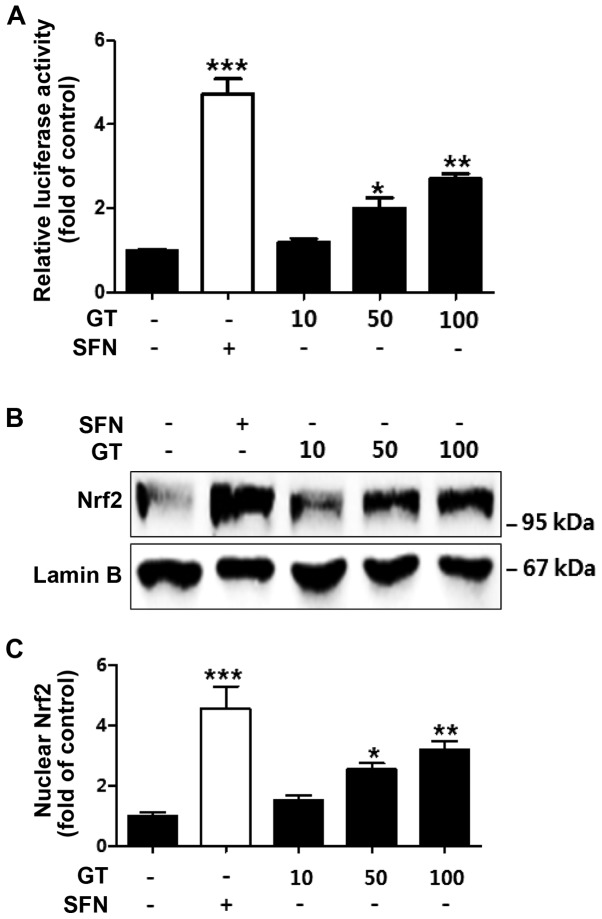
*Geranium thunbergii* (GT) enhances the transcriptional activity and nuclear translocation of nuclear factor erythroid 2-related factor 2 (Nrf2) in RAW264.7 cells. (A) An Nrf2 reporter cell line was treated with sulforaphane (SFN; 5 *μ*M) or the indicated amounts of GT for 16 h. Luciferase activity was normalized by the amount of total proteins. ^*^p<0.05, ^**^p<0.01 and ^***^p<0.001 compared to the control (first column). All data represent the means ± SD of 3 independent experiments. (B) The cells were treated with the indicated concentrations of GT for 8 h. SFN (5 *μ*M) was used as a positive control. The amount of nuclear Nrf2 was estimated by western blot analysis. The amount of lamin B was used as an internal control. (C) The intensity of the bands was densitometrically quantified and calculated as the means ± SD of 3 independent experiments. ^*^p<0.05, ^**^p<0.01 and ^***^p<0.001 compared to the control (first column).

To elucidate the mechanisms responsible for the anti-inflammatory effects of GT, we first examined the suppressive effects of GT on the INF-γ- and LPS-induced NF-κB activity. Of note, the transcriptional activity of NF-κB, measured by luciferase assay using NF-κB reporter cells, was not reduced by treatment with GT (Fig. 3A). The nuclear translocation of NF-κB was also not altered (Fig. 3B and C). However, GT induced the transcriptional activity of Nrf2 in a dose-dependent manner (Fig. 4A). The nuclear translocation of Nrf2 was also significantly increased by treatment with GT in a dose-dependent manner (Fig. 4B and C).

**Figure 5:**
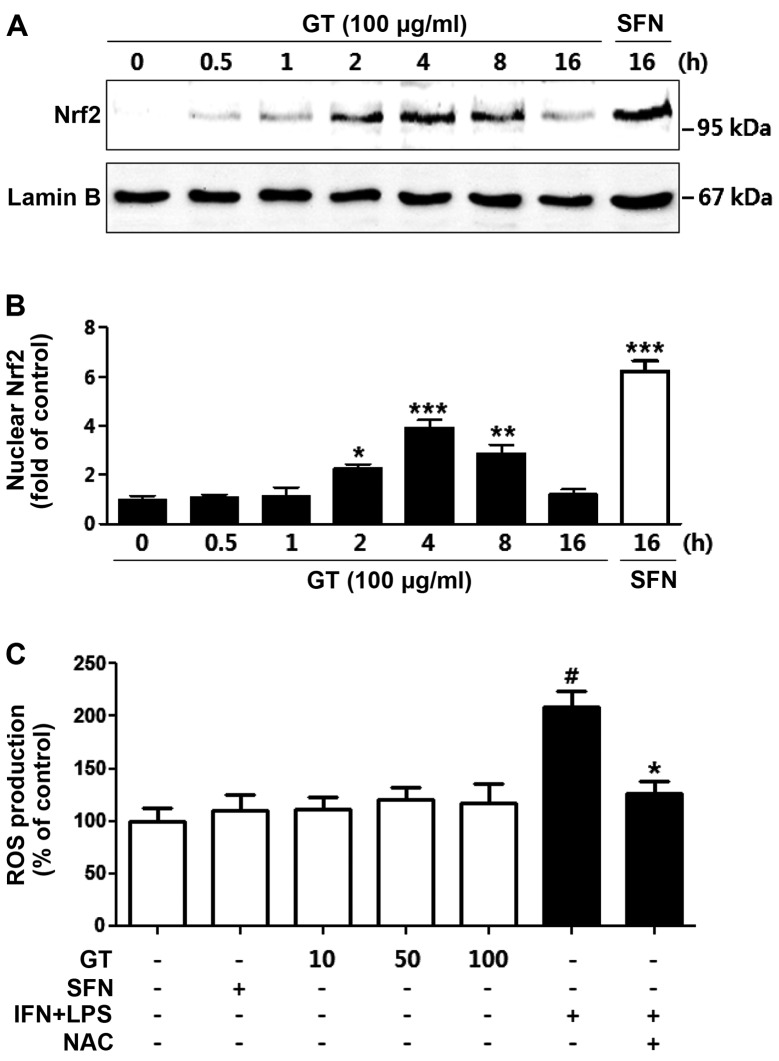
Effect of *Geranium thunbergii* (GT) on the time-dependent nuclear translocation of nuclear factor erythroid 2-related factor 2 (Nrf2) and production of intracellular reactive oxygen species (ROS). (A) The RAW264.7 cells were treated with 100 *μ*g/ml of GT for the indicated periods of time. The amount of nuclear Nrf2 was examined by western blot analysis. The amount of lamin B was used as an internal control. (B) The intensity of the bands was densitometrically quantified and calculated as the means ± SEM of 3 independent experiments. ^*^p<0.05, ^**^p<0.01 and ^***^p<0.001 compared to the control (first column). (C) The RAW264.7 cells were treated with the indicated concentrations of GT or sulforaphane (SFN; 5 *μ*M) for 16 h. The cells treated with interferon-γ (IFN-γ) and lipopolysaccharides (LPS) (10 ng/ml, respectively) and/or N-acetyl cysteine (NAC) (1 mM; used as a control). The production of intracellular ROS was estimated using carboxy-H2DCFDA. The data from 3 independent experiments are represented as the means ± SD. ^#^p<0.01 compared to the negative control (first column); ^*^p<0.05 compared to the positive control (sixth column).

The time-dependent activation of Nrf2 was determined by detecting the nuclear translocation of Nrf2 by western blot analysis. The results revealed that the nuclear translocation of Nrf2 was significantly increased at 2 to 8 h of treatment with 100 *μ*g/ml of GT (Fig. 5A and B). In order to exclude the possibility that ROS activates Nrf2 to protect the cells from oxidative damage (27), we examined the production of ROS by the GT-treated RAW264.7 cells. GT did not significantly increase ROS production. Taken together, these results indicate that the GT-induced transcriptional activity of Nrf2 is not mediated by ROS (Fig. 5C).

### GT enhances the expression of Nrf2-dependent genes

**Figure 6:**
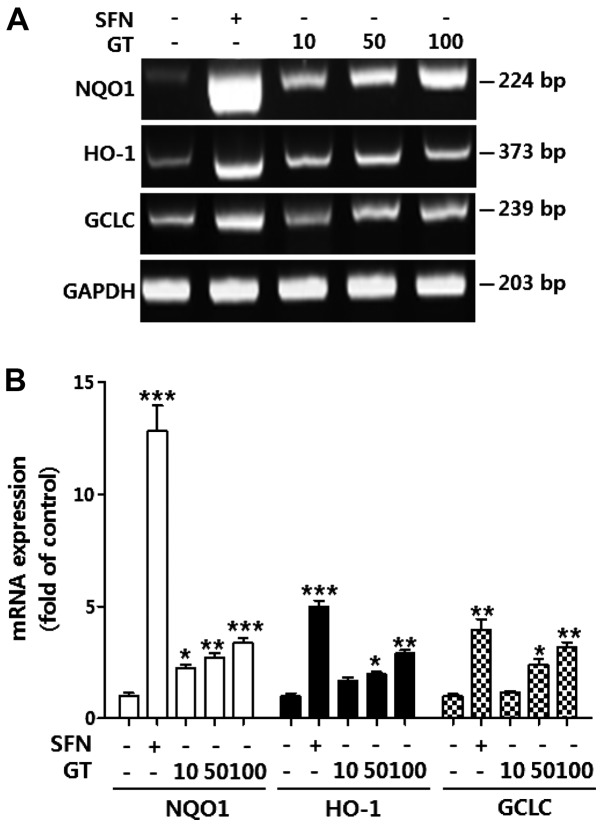
*Geranium thunbergii* (GT) enhances the expression of nuclear factor erythroid 2-related factor 2 (Nrf2)-dependent genes. (A) The RAW264.7 cells were treated with the indicated concentrations of GT for 16 h. sulforaphane (SFN; 5 *μ*M) was used as a positive control. The expression of Nrf2-dependent genes, including NAD(P)H: quinine oxidoreductase 1 (*NQO1*), heme oxygenase-1 (*HO-1*) and glutamate-cysteine ligase catalytic subunit (*GCLC*), were evaluated by RT-PCR. The expression of glyceraldehyde-3-phosphate dehydrogenase (*GAPDH*) was used as an internal control. (B) The intensity of the bands was densitometrically quantified and calculated as the means ± SD of 3 independent experiments. ^*^p<0.05, ^**^p<0.01 and ^***^p<0.001 compared to the control (first column).

The expression of Nrf2-dependent antioxidant genes may contribute to the suppression of inflammatory responses (28). Thus, we examined the expression levels of Nrf2-dependent genes, including *NQO1*, *HO-1* and *GCLC*. The results revealed a dose-dependent increase in the expression of Nrf2-dependent genes, which positively correlated with Nrf2 activity (Fig. 6). The expression levels of Nrf2-dependent genes were increased by 3.4-fold for *NQO1*, 2.8-fold for *HO-1* and by 3.1-fold for *GCLC* when the cells were treated with 100 *μ*g/ml of GT.

### The anti-inflammatory effects of GT are Nrf2-dependent

**Figure 7:**
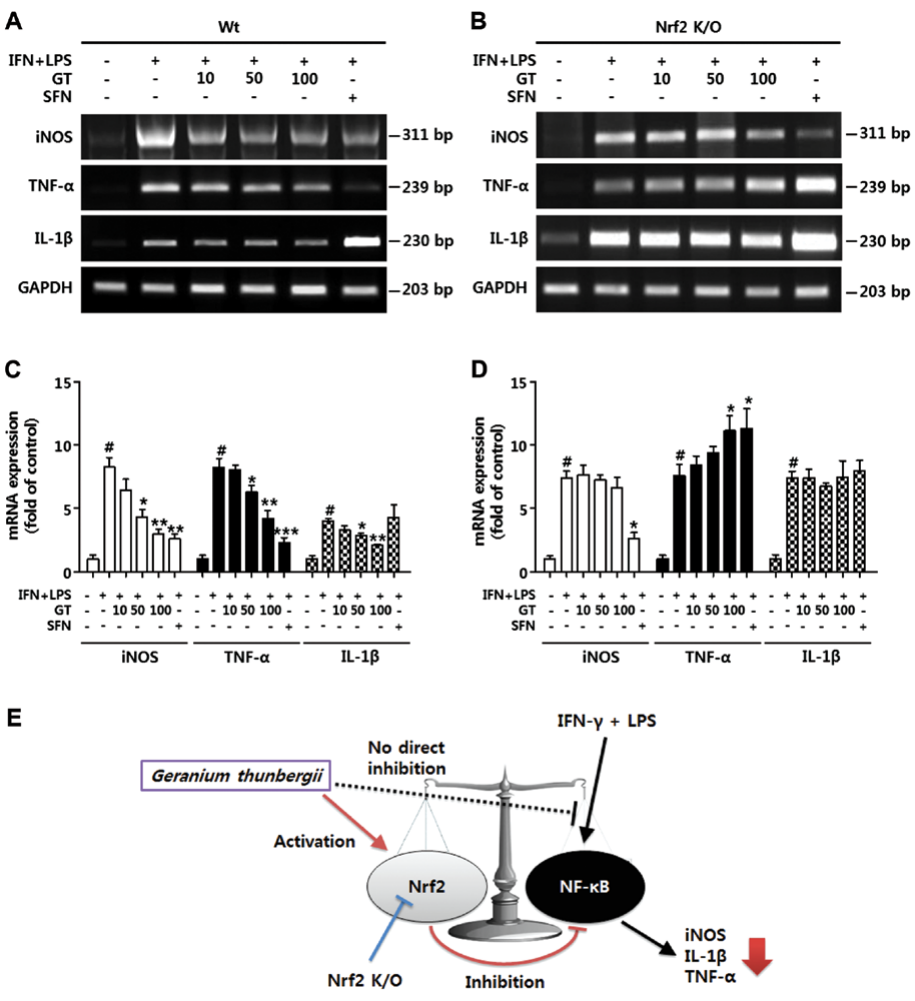
Inhibitory effects of *Geranium thunbergii* (GT) on the expression of pro-inflammatory genes are regulated by nuclear factor erythroid 2-related factor 2 (Nrf2) activity. (A and B) Bone marrow-derived macrophages from (A) wild-type C57BL/6 or (B) Nrf2 knockout mice were treated with GT (100 *μ*g/ml) or sulforaphane (SFN; 5 *μ*M) for 4 h prior to stimulation with interferon-γ (IFN-γ) and lipopolysaccharides (LPS) (10 ng/ml, respectively). The expression of pro-inflammatory genes, including inducible nitric oxide synthase (*iNOS)*, interleukin-1β (*IL-1β*) and tumor necrosis factor-α (*TNF-α*) were estimated by RT-PCR. The expression of glyceraldehyde-3-phosphate dehydrogenase (*GAPDH*) was used as an internal control. (C and D) The intensity of the bands was densitometrically quantitated and calculated as the mean ± SD of 3 independent experiments. ^#^p<0.001 compared to the negative control (first column); ^*^p<0.05, ^**^p<0.01 and ^***^p<0.001 compared to the positive control (second column). (E) A schematic representation of the anti-inflammatory effects of GT.

Subsequently, we investigated the possibility that Nrf2 activation has a direct association with the anti-inflammatory effects of GT. The macrophages derived from the bone marrow of wild-type C57BL/6 mice showed a similar response to GT treatment. Namely, the expression of NF-κB-dependent pro-inflammatory genes was significantly reduced by treatment with GT in a dose-dependent manner (Fig. 7A and C). However, in the macrophages derived from the bone marrow of Nrf2 knockout mice, the expression of NF-κB-dependent genes was not diminished. On the contrary, the expression of TNF-α was increased in accordance with the concentration of GT (Fig. 7B and D). These results clearly indicate that the anti-inflammatory effects of GT are related to Nrf2 activity.

## Discussion

The regulation of macrophage activation is important for the initiation and resolution of inflammation (2). Classically activated macrophages are induced by combined stimulation with LPS and IFN-γ; these macrophages express a unique set of genes, including *TNF-α*, *IL-1β*, *IL-6*, cyclooxygenase-2 (*COX-2*) and *iNOS* (29). Thus, the combined stimulation of macrophages with LPS and IFN-γ is used to create *in vitro* models of acute and chronic inflammatory conditions, including septic shock, rheumatoid arthritis, inflammatory bowel disease and chronic obstructive pulmonary disease (30).

The high production of cytokines, such as TNF-α, IL-1β is considered to be a major factor involved in the induction and maintenance of inflammation (29). In addition, iNOS serves as a pro-inflammatory agent by producing nitric oxide, a mediator of the inflammatory response (31). The expression of these genes is mainly regulated by NF-κB; thus the classical strategy for the development of therapeutic agents against inflammation has focused on the suppression of NF-κB activity (32).

In the present study, we demonstrated that GT, a medicinal herb used for the treatment of diarrhea and dysentery (16), exerted an inhibitory effect on LPS- and IFN-γ-induced inflammation at non-toxic doses (Figs. 1 and 2). Firstly, it was assumed that the anti-inflammatory effects of GT on LPS- and IFN-γ-stimulated macrophages may be due to the suppression of NF-κB. However, surprisingly to us, GT did not reduce the transcriptional activity or nuclear translocation of NF-κB (Fig. 3).

As NF-κB also has many beneficial effects on organisms, including protection against infection, the activation of the immune response and the regulation of energy metabolism (33,34), researchers have focused on Nrf2 as an alternative molecular target for suppressing inflammation (6,35,36). The disruption of Nrf2 using a knockout technique has been shown to enhance NF-κB activation in diverse disease models (36,37). In general, the Nrf2-mediated anti-inflammatory effects are thought to be achieved through the activation of antioxidant enzymes and the consequent suppression of the ROS-NF-κB signaling pathway, but not through direct interaction between Nrf2 and NF-κB (38,39). Thus, we also examined the effect of GT on Nrf2 activity. The results revealed that GT enhanced Nrf2 activity without inducing significant cytotoxicity or ROS production (Figs. 4 and 5). In addition, the expression of Nrf2-dependent antioxidant genes, including *NQO1*, *HO-1* and *GCLC* were also increased by treatment with GT (Fig. 6). From these results, it was can by hypothesized that the inhibitory effects of GT on IFN-γ- and LPS-induced inflammatory responses may be related to Nrf2 activation.

To confirm this possibility, we examined the suppressive effects of GT on the expression of NF-κB-dependent genes in BMDM isolated from Nrf2 knockout mice. In BMDM from Nrf2 knockout mice, the mRNA levels of NF-κB-dependent genes, including *TNF-α*, *IL-1β* and *iNOS*, were not reduced by treatment with GT (Fig. 7A–D). Taken together, these data indicate that the inhibitory effects of GT on IFN-γ- and LPS-induced inflammation are related, at least in part, to the activation of Nrf2 (Fig. 7E). Most Nrf2 activators, such as caffeic acid phenethyl ester (40), curcumin (41), quercetin (42) and SFN (43), have dual functions; they activate Nrf2 and inhibit NF-κB. Unlike in previous studies, our results demonstrated that GT does not directly affect NF-κB activity (Fig. 3A–C). Other previous studies (13,25,44,45) have also demonstrated that the Nrf2-dependent activation of antioxidant systems reduces inflammation without the suppression of NF-κB. The present study had the limitation of addressing Nrf2 activation in a cell type specific manner, specifically, in macrophages. Nevertheless, it is obvious that the inhibitory effects of GT on IFN-γ- and LPS-induced inflammation are dependent on Nrf2 activation. In addition, our results suggest that GT contains lead compounds which may be used for the effective treatment of macrophage-mediated inflammation.

In this study, to the best of our knowledge, we provide the first experimental evidence that GT exerts inhibitory effects on LPS- and IFN-γ-induced inflammation by enhancing Nrf2 activity without directly affecting NF-κB activity. These results suggest that the therapeutic effects of GT in traditional usage for various inflammatory diseases are, at least in a part, associated with the activation of Nrf2, a key transcription factor in anti-inflammatory systems. Our results also raise the possibility that GT may be used as a candidate agent for the further development of novel and specific therapies aiming at promoting Nrf2 activation.

## References

[1] Reuter S, Gupta SC, Chaturvedi MM, Aggarwal BB (2010). Oxidative stress, inflammation, and cancer: How are they linked?. Free Radic Biol Med.

[2] Nathan C, Ding A (2010). Nonresolving inflammation. Cell.

[3] Laskin DL, Sunil VR, Gardner CR, Laskin JD (2011). Macrophages and tissue injury: Agents of defense or destruction?. Annu Rev Pharmacol Toxicol.

[4] Davignon JL, Hayder M, Baron M, Boyer JF, Constantin A, Apparailly F, Poupot R, Cantagrel A (2013). Targeting monocytes/macrophages in the treatment of rheumatoid arthritis. Rheumatology (Oxford).

[5] Alam J, Stewart D, Touchard C, Boinapally S, Choi AM, Cook JL (1999). Nrf2, a Cap‘n’Collar transcription factor, regulates induction of the heme oxygenase-1 gene. J Biol Chem.

[6] Baird L, Dinkova-Kostova AT (2011). The cytoprotective role of the Keap1-Nrf2 pathway. Arch Toxicol.

[7] Maicas N, Ferrándiz ML, Brines R, Ibáñez L, Cuadrado A, Koenders MI, van den Berg WB, Alcaraz MJ (2011). Deficiency of Nrf2 accelerates the effector phase of arthritis and aggravates joint disease. Antioxid Redox Signal.

[8] Khor TO, Huang MT, Kwon KH, Chan JY, Reddy BS, Kong AN (2006). Nrf2-deficient mice have an increased susceptibility to dextran sulfate sodium-induced colitis. Cancer Res.

[9] Chan K, Kan YW (1999). Nrf2 is essential for protection against acute pulmonary injury in mice. Proc Natl Acad Sci USA.

[10] Kaplan M, Mutlu EA, Benson M, Fields JZ, Banan A, Keshavarzian A (2007). Use of herbal preparations in the treatment of oxidant-mediated inflammatory disorders. Complement Ther Med.

[11] Lee SE, Jeong SI, Yang H, Jeong SH, Jang YP, Park CS, Kim J, Park YS (2012). Extract of Salvia miltiorrhiza (Danshen) induces Nrf2-mediated heme oxygenase-1 expression as a cytoprotective action in RAW 264.7 macrophages. J Ethnopharmacol.

[12] Lee JA, Lee MY, Shin IS, Seo CS, Ha H, Shin HK (2012). Anti-inflammatory effects of Amomum compactum on RAW 264.7 cells via induction of heme oxygenase-1. Arch Pharm Res.

[13] Choi JY, Kwun MJ, Kim KH (2012). Protective Effect of the fruit hull of Gleditsia sinensis on LPS-induced acute lung injury is associated with Nrf2 activation. Evid Based Complement Alternat Med.

[14] Han CW, Kwun MJ, Kim KH, Choi JY, Oh SR, Ahn KS, Lee JH, Joo M (2013). Ethanol extract of Alismatis Rhizoma reduces acute lung inflammation by suppressing NF-κB and activating Nrf2. J Ethnopharmacol.

[15] Ju MS, Jeong HU, Kim HG (2010). Anti-nociceptive and anti-inflammatory effects of Geranii Herba. Kor J Herbol.

[16] Liu QH, Jeong JE, Choi EJ, Moon YH, Woo ER (2006). A new furofuran lignan from Geranium thunbergii Sieb. et Zucc. Arch Pharm Res.

[17] Hiramatsu N, Xiufen W, Takechi R, Itoh Y, Mamo J, Pal S (2004). Antimutagenicity of Japanese traditional herbs, gennoshoko, yomogi, senburi and iwa-tobacco. Biofactors.

[18] Xiufen W, Hiramatsu N, Matsubara M (2004). The antioxidative activity of traditional Japanese herbs. Biofactors.

[19] Sung YY, Yoon T, Yang WK, Kim SJ, Kim HK (2011). Anti-obesity effects of Geranium thunbergii extract via improvement of lipid metabolism in high-fat diet-induced obese mice. Mol Med Rep.

[20] Liu QH, Woo ER (2008). Inhibitory activity of IL-6 production by flavonoids and phenolic compounds. Nat Prod Sci.

[21] Okuda T, Mori K, Murakami R (1977). Constituents of Geranium thunbergii Sieb. et Zucc. VI. Difference of tannin activity caused by structural differences. (2). Colorimetry with methylene blue (author’s transl). Yakugaku Zasshi.

[22] Okuda T, Yoshida T, Mori K (1975). Consitutents of Geranium thunbergii Sieb. et Zucc. II. Ellagitannins. (1) (author’s transl). Yakugaku Zasshi.

[23] Harborne JB, Baxter H, Moss GP (1999). Phytochemical Dictionary: A Handbook of Bioactive Compounds from Plants.

[24] Barthwal MK, Anzinger JJ, Xu Q, Bohnacker T, Wymann MP, Kruth HS (2013). Fluid-phase pinocytosis of native low density lipoprotein promotes murine M-CSF differentiated macrophage foam cell formation. PLoS One.

[25] Lyu JH, Lee GS, Kim KH (2011). ent-kaur-16-en-19-oic Acid, isolated from the roots of Aralia continentalis, induces activation of Nrf2. J Ethnopharmacol.

[26] Okuda T, Mori K, Ishino M (1979). Constituents of Geranium thunbergii Sieg. et Zucc. VIII. Transformations of geraniin upon decoction (author’s transl). Yakugaku Zasshi.

[27] Johnson JA, Johnson DA, Kraft AD, Calkins MJ, Jakel RJ, Vargas MR, Chen PC (2008). The Nrf2-ARE pathway: An indicator and modulator of oxidative stress in neurodegeneration. Ann NY Acad Sci.

[28] Wakabayashi N, Slocum SL, Skoko JJ, Shin S, Kensler TW (2010). When NRF2 talks, who’s listening?. Antioxid Redox Signal.

[29] Drexler SK, Kong PL, Wales J, Foxwell BM (2008). Cell signalling in macrophages, the principal innate immune effector cells of rheumatoid arthritis. Arthritis Res Ther.

[30] Schroder K, Sweet MJ, Hume DA (2006). Signal integration between IFNgamma and TLR signalling pathways in macrophages. Immunobiology.

[31] Korhonen R, Lahti A, Kankaanranta H, Moilanen E (2005). Nitric oxide production and signaling in inflammation. Curr Drug Targets Inflamm Allergy.

[32] Gupta SC, Sundaram C, Reuter S, Aggarwal BB (2010). Inhibiting NF-κB activation by small molecules as a therapeutic strategy. Biochim Biophys Acta.

[33] Tornatore L, Thotakura AK, Bennett J, Moretti M, Franzoso G (2012). The nuclear factor-kappa B signaling pathway: Integrating metabolism with inflammation. Trends Cell Biol.

[34] Spehlmann ME, Eckmann L (2009). Nuclear factor-kappa B in intestinal protection and destruction. Curr Opin Gastroenterol.

[35] Saito H (2013). Toxico-pharmacological perspective of the Nrf2-Keap1 defense system against oxidative stress in kidney diseases. Biochem Pharmacol.

[36] Kim J, Cha YN, Surh YJ (2010). A protective role of nuclear factor-erythroid 2-related factor-2 (Nrf2) in inflammatory disorders. Mutat Res.

[37] Jin W, Wang H, Yan W, Xu L, Wang X, Zhao X, Yang X, Chen G, Ji Y (2008). Disruption of Nrf2 enhances upregulation of nuclear factor-kappaB activity, proinflammatory cytokines, and inter-cellular adhesion molecule-1 in the brain after traumatic brain injury. Mediators Inflamm.

[38] Turpaev KT (2013). Keap1-Nrf2 signaling pathway: Mechanisms of regulation and role in protection of cells against toxicity caused by xenobiotics and electrophiles. Biochem Mosc.

[39] Jeong WS, Kim IW, Hu R, Kong AN (2004). Modulatory properties of various natural chemopreventive agents on the activation of NF-kappaB signaling pathway. Pharm Res.

[40] Márquez N, Sancho R, Macho A, Calzado MA, Fiebich BL, Muñoz E (2004). Caffeic acid phenethyl ester inhibits T-cell activation by targeting both nuclear factor of activated T-cells and NF-kappaB transcription factors. J Pharmacol Exp Ther.

[41] Xu YX, Pindolia KR, Janakiraman N, Chapman RA, Gautam SC (1997–1998). Curcumin inhibits IL1 alpha and TNF-alpha induction of AP-1 and NF-κB DNA-binding activity in bone marrow stromal cells. Hematopathol Mol Hematol.

[42] Moreira AJ, Fraga C, Alonso M, Collado PS, Zetller C, Marroni C, Marroni N, González-Gallego J (2004). Quercetin prevents oxidative stress and NF-kappaB activation in gastric mucosa of portal hypertensive rats. Biochem Pharmacol.

[43] Xu C, Shen G, Chen C, Gélinas C, Kong AN (2005). Suppression of NF-kappaB and NF-kappaB-regulated gene expression by sulforaphane and PEITC through IkappaBalpha, IKK pathway in human prostate cancer PC-3 cells. Oncogene.

[44] Manandhar S, You A, Lee ES, Kim JA, Kwak MK (2008). Activation of the Nrf2-antioxidant system by a novel cyclooxygenase-2 inhibitor furan-2-yl-3-pyridin-2-yl-propenone: Implication in anti-inflammatory function by Nrf2 activator. J Pharm Pharmacol.

[45] Li B, Abdalrahman A, Lai Y, Janicki JS, Ward KW, Meyer CJ, Wang XL, Tang D, Cui T (2014). Dihydro-CDDO-trifluoroethyl amide suppresses inflammatory responses in macrophages via activation of Nrf2. Biochem Biophys Res Commun.

